# Tert-Butylhydroquinone Prevents Oxidative Stress-Mediated Apoptosis and Extracellular Matrix Degradation in Rat Chondrocytes

**DOI:** 10.1155/2021/1905995

**Published:** 2021-12-08

**Authors:** Bo Yang, Haisheng Huang, Qisong He, Wei Lu, Lu Zheng, Li Cui

**Affiliations:** ^1^Department of Orthopedic Surgery, Shanghai TCM-Integrated Hospital Shanghai University of TCM, No. 230, Baoding Road, Hongkou, Shanghai, China; ^2^Department of Acupuncture, Shanghai TCM-Integrated Hospital Shanghai University of TCM, No. 230, Baoding Road, Hongkou, Shanghai, China

## Abstract

Oxidative stress-induced chondrocyte apoptosis and degradation of the extracellular matrix (ECM) play an important role in the progression of osteoarthritis (OA). In addition, tert-butylhydroquinone (TBHQ) is an activator of the nuclear factor erythroid derived-2-related factor 2 (Nrf2). The present study aimed to determine the effectiveness of TBHQ in preventing the apoptosis of chondrocytes and degradation of the extracellular matrix, induced by oxidative stress, in vitro. Therefore, rat chondrocytes were exposed to 20 *μ*M tert-butyl hydroperoxide (TBHP) for 24 h to establish an oxidative damage model, in vitro. Thereafter, cell viability was evaluated using the Cell Counting Kit-8 assay. Moreover, the level of ROS was determined through 2′,7′-dichlorofluorescein diacetate staining. The mitochondrial membrane potential of chondrocytes was also measured using JC-1. Furthermore, cell apoptosis was assessed through Annexin V-fluorescein isothiocyanate/propidium iodide staining. The study also performed Western blotting and qPCR to evaluate the expression of extracellular matrix components, matrix catabolic enzymes, and changes in signalling pathways. The results showed that 2.5 and 5 *μ*M of TBHQ reduced the TBHP-induced generation of excessive ROS and improved cell viability. Additionally, 2.5 and 5 *μ*M of TBHQ prevented mitochondrial damage and apoptosis in rat chondrocytes. Treatment with TBHQ also increased the mRNA and protein expression levels of aggrecan and collagen II. However, TBHQ reduced the mRNA and protein expression levels of matrix metalloproteinase 3 (MMP3) and matrix metalloproteinase 13 (MMP13) in rat chondrocytes. In addition, treatment with TBHQ enhanced the protein expression levels of Nrf2, NADPH quinone oxidoreductase 1 (NQO-1), and hemeoxygenase-1 (HO-1) in rat chondrocytes. The current study showed that TBHQ was not only effective in protecting against TBHP-induced oxidative stress but also inhibited the apoptosis of rat chondrocytes and degradation of the ECM by activating the Nrf2 pathway. The results therefore suggest that TBHQ holds potential for use in the treatment of OA.

## 1. Introduction

Osteoarthritis (OA) is the most common form of arthritis that not only severely affects the patients' quality of life but also causes a substantial clinical and socioeconomic burden. OA affects more than 500 million people worldwide. From 1990 to 2019, the number of people globally suffering from OA increased by 48%. The pathological changes in OA include cartilage degeneration, synovial inflammation, and subchondral bone thickening. Currently, nonsteroidal anti-inflammatory drugs (NSAIDs) and tramadol are recommended for the clinical treatment of OA. However, both NSAIDs and tramadol are only used for the short-term relief of symptoms. Safe and effective drugs for preventing or reversing the progression of OA are therefore still lacking [1–3].

Notably, chondrocytes are the only population of cells existing in healthy cartilage. The cells are involved in regulating the growth, distribution, and reconstruction of the cartilage matrix. In addition, the increased apoptosis of chondrocytes and the subsequent degradation of the extracellular matrix (ECM), caused by multiple factors, are considered to be signs of cartilage degradation in OA [4–6]. Numerous studies have also shown that oxidative stress plays an important role in the occurrence and progression of OA. Moreover, excessive generation of reactive oxygen species induces oxidative damage in chondrocytes, which leads to chondrocyte apoptosis and degradation of the ECM. This in turn causes irreversible damage to the articular cartilage, which contributes to the progression of OA [7–9]. Therefore, inhibiting the oxidative stress-induced apoptosis of chondrocytes and ECM degradation may be an effective means of preventing cartilage degeneration and progression of OA.

The nuclear factor erythroid derived-2-related factor 2 (Nrf2) is a major regulatory factor for antioxidant proteins. Under normal circumstances, the Kelch-like ECH-associated protein 1 (Keap1) binds to Nrf2 in the cytoplasm, hence, inhibiting the activation of Nrf2. However, stimulation through exposure to excessive ROS causes the disassociation of Nrf2-Keap1 and accumulation of Nrf2 in the nucleus. Thereafter, Nrf2 binds to the consensus sequences of the antioxidant response element (ARE), promoting the expression of antioxidant and phase 2 defense enzymes, including hemeoxygenase‐1 (HO‐1) and NADPH quinone oxidoreductase 1 (NQO-1) [10]. Moreover, the Nrf2 pathway is known to be associated with oxidative stress and may therefore be a potential pharmacological target for oxidative disorders [11–13].

Furthermore, tert-butylhydroquinone (TBHQ) is an aromatic organic compound that has been used for a long time as a food preservative. It was also reported that TBHQ is one of the most potent activators of the Nrf2/Keap1/ARE signalling pathway [14]. Additionally, TBHQ was proven to have strong antioxidant effects through the activation of the Nrf2 pathway, in in vitro or in vivo studies of some diseases such as alcoholic cardiomyopathy, acute hepatic injury, and neurodegenerative disease [15–18]. However, it is still unknown whether TBHQ can inhibit the apoptosis of chondrocytes and ECM degradation, induced by oxidative stress. Therefore, the present study attempted to investigate the ability of TBHQ to inhibit the apoptosis of chondrocytes and ECM degradation, induced by oxidative stress. The possible underlying mechanisms were also assessed.

## 2. Materials and Methods

### 2.1. Cell Isolation and Culture

All the animal experiments were approved by the ethics committee on animal experiments of Shanghai TCM-Integrated Hospital, Shanghai University of TCM (Shanghai, China). In this study, three 4-week-old male Sprague-Dawley rats were euthanized through inhalation of CO_2_, and death was confirmed by the presence of cardiac and respiratory arrest. Thereafter, the rat cartilages were separated from the bilateral hip joints and digested with a 0.25% trypsin (Beyotime Institute of Biotechnology, Shanghai, China) solution for 1 h, followed by overnight digestion with a 0.1% collagenase II solution (Sigma-Aldrich; Merck KGaA, St. Louis, MO, USA). The isolated chondrocytes were then collected and cultured in Dulbecco's modified Eagle's medium (DMEM; Gibco; Grand Island, NY, USA) supplemented with 10% foetal bovine serum (FBS; Gibco) at 37°C and 5% CO_2_.

The chondrocytes were passaged for the subsequent in vitro studies when the cells reached 80–90% confluence. Moreover, the oxidative stress cell model was induced using tert‐butyl hydroperoxide (TBHP), whose concentration (20 *μ*M) was selected based on previous literature [19].

### 2.2. Cell Viability Assay

Cell viability was measured using the Cell Counting Kit-8 (CCK-8) assay (Beyotime). Briefly, the cells were seeded in 96-well plates at a density of 1 × 10^4^ cells/well. Thereafter, the cells were treated with 20 *μ*M TBHP and different concentrations of TBHQ for 24 h. After treatment, the cells were incubated with 10 *μ*l of the CCK-8 solution added in 100 *μ*l of serum-free DMEM, at 37°C for 2 h. Afterwards, absorbance was evaluated at 450 nm using a microplate reader (Epoch; BioTek Instruments, Inc.).

### 2.3. Quantification of ROS Production

The levels of ROS in chondrocytes were evaluated using the ROS sensitive dye, 2′,7′-dichlorodihydrofluorescein diacetate (DCFH-DA; Sigma-Aldrich). Briefly, chondrocytes were collected and washed thrice with phosphate-buffered saline (PBS, Gibco). Thereafter, the cells were stained with 10 *μ*M of DCFH-DA for 30 min. Afterwards, the cells were washed thrice with a serum-free medium to remove the residual extracellular DCFH-DA. Finally, the levels of ROS were evaluated using the BD Accuri C6 Plus flow cytometer (BD Biosciences, Vianen, The Netherlands).

### 2.4. Detection of Mitochondrial Membrane Potential

The mitochondrial membrane potential of chondrocytes was assessed using a JC-1 staining kit (Beyotime). Briefly, the cells were collected and washed three times with PBS. Thereafter, the cells were stained with JC-1 (5 *μ*g/ml) for 20 min at 37°C. The cells were then washed again three times with PBS to remove the residual JC-1. Cells were observed under confocal microscopy (Olympus, FV3000) (magnification, ×200). In addition, the mitochondrial membrane potential of chondrocytes was evaluated using the BD Accuri C6 Plus flow cytometer, and the data were analyzed using the FlowJo software (FlowJo LLC, version 10.6.0).

### 2.5. Determination of Cell Apoptosis

Annexin V-fluorescein isothiocyanate (FITC)/propidium iodide (PI) staining (BD Biosciences) was used to determine chondrocyte apoptosis. Briefly, the cells were collected and washed thrice with ice-cold PBS. Thereafter, they were incubated with 5u Annexin V-FITC added in 300 *μ*l of binding buffer for 25 min and then stained with 5 *μ*l of PI added in 200 *μ*l of binding buffer for 5 min, at room temperature in the dark. Chondrocyte apoptosis was then evaluated within 30 min using the BD Accuri C6 Plus flow cytometer, and the data were analyzed using the FlowJo software.

### 2.6. Determination of Malondialdehyde (MDA) and Superoxide Dismutase (SOD) Levels

The levels of superoxide dismutase (SOD) and malondialdehyde (MDA) in chondrocytes were determined using the SOD and MDA assay kits (Beyotime), according to the manufacturer's instructions. Briefly, the cell lysates were treated with a working buffer for 30 min at 37°C. Absorbance was then measured at a wavelength of 523 nm (MDA) or 450 nm (SOD) using a microplate reader. Additionally, total protein concentration was measured using a bicinchoninic acid (BCA) protein assay kit (Thermo Fisher, Waltham, MA, USA) to normalize the MDA and SOD levels.

### 2.7. Real-Time PCR Assay

Total RNA was extracted from chondrocytes using the RNA simple total RNA kit (Tiangen Biotech, Beijing, China). Thereafter, single-stranded cDNA was synthesized from 1000 ng of the extracted RNA using a reverse transcript master mix (TaKaRa, Shiga, Japan). Afterwards, quantitative RT-PCR was performed using the PowerUp SYBR Green Master Mix on the QuantStudio 5 real-time PCR system (Applied Biosystems, Foster City, CA, USA). Moreover, the expression of GAPDH was used to normalize the Ct values. The forward and reverse primer sequences used in this study are given in Table 1.

### 2.8. Western Blotting

After treatment, the chondrocytes were lysed in the radioimmunoprecipitation assay buffer to extract total protein. Equal amounts of protein were then separated through 12% sodium dodecyl sulfate-polyacrylamide gel electrophoresis (SDS-PAGE), after which they were transferred onto nitrocellulose membranes (Invitrogen, Carlsbad, CA, USA). The membranes were then incubated overnight at 4°C with the following antibodies: Nrf2 (1 : 1,000; Proteintech, Wuhan, China), NQO-1 (1 : 5,000; Proteintech), HO-1 (1 : 1,000; Proteintech), aggrecan (1 : 1,000; Abcam, Cambridge, MA, USA), collagen II (1 : 1,000; Abcam), cleaved caspase-3 (1 : 1,000; CST), glyceraldehyde 3-phosphate dehydrogenase (GAPDH, 1 : 2,000; Cell Signalling Technology Inc., Danvers, MA, USA), matrix metalloproteinase 13 (MMP13, 1 : 1,000; CST), and MMP 3 (1 : 1,000; CST). Afterwards, the membranes were washed with TSB-Tween (0.05%) for 30 min, followed by incubation with the anti-rabbit secondary antibody (1 : 5,000; CST) for 2 h, at room temperature. Finally, the protein signals were visualized using an enhanced chemiluminescence (ECL) detection reagent and then imaged using the G:BOX ChemiXR5 imaging system.

### 2.9. Statistical Analysis

Each experiment was independently performed at least three times. Statistical comparisons among multiple groups were then performed using one-way ANOVA followed by Bonferroni's multiple comparison test, in the SPSS software (version 20.0; ITBHQ Corp.). In addition, data were expressed as the mean ± standard deviation, and statistical significance was set at *p* < 0.05.

## 3. Results

### 3.1. Effects of TBHQ on the Viability of Rat Chondrocytes

**Figure 1:**
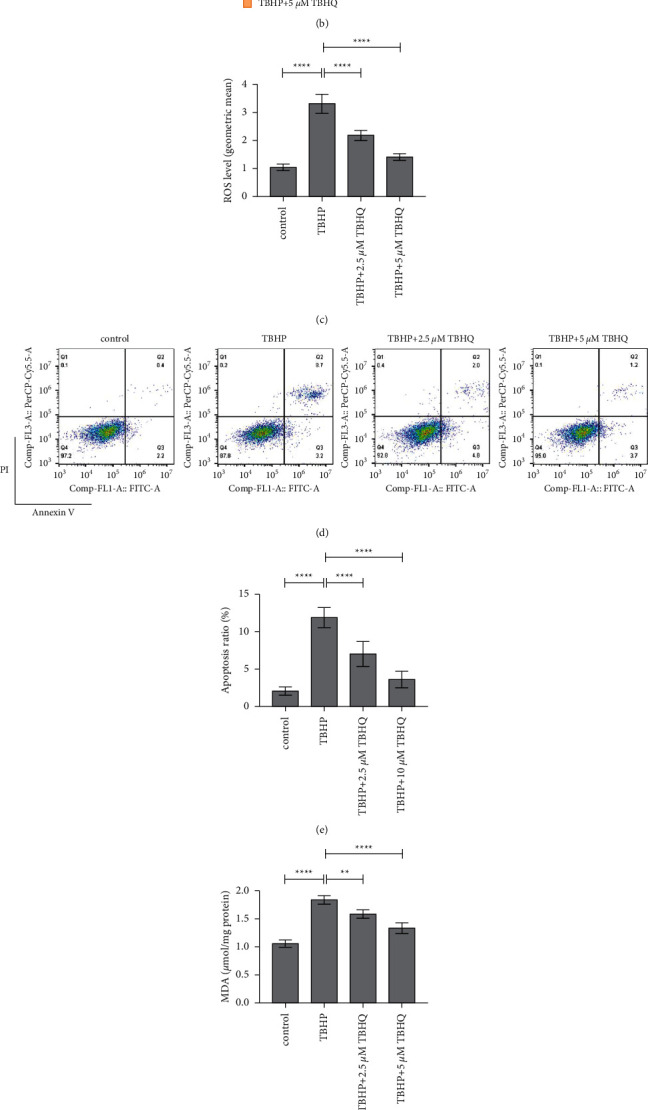
TBHQ prevented excessive generation of ROS, increased the levels of SOD, and decreased the levels of MDA, and promoted cell apoptosis in rat chondrocytes treated with 20 *μ*M TBHP. (a) The structural formula of TBHQ. (b-c) Chondrocytes stained with DCFH-DA and then quantified through flow cytometry to determine the levels of ROS. The results showed that 2.5 and 5 *μ*M of TBHQ effectively prevented excessive generation of ROS in rat chondrocytes treated with 20 *μ*M THBP. (d-e) Annexin V/PI staining was used to determine the apoptosis of rat chondrocytes. Flow cytometry analysis showed that treatment with 2.5 and 5 *μ*M  of TBHQ significantly reduced the rate of apoptosis in chondrocytes treated with 20 *μ*M  TBHP. (f-g) 2.5 and 5 *μ*M  of TBHQ significantly decreased the levels of MDA and increased the levels of SOD in rat chondrocytes treated with 20 *μ*M THBP. ^*∗∗*^*P* < 0.05 and ^*∗∗∗∗*^*P* < 0.01.

**Figure 2:**
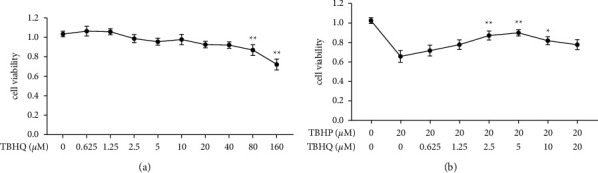
TBHQ improved the viability of rat chondrocytes treated with 20 *μ*M  TBHP. (a) Effects of different concentrations (0, 0.625, 1.25, 2.5, 5, 10, 20, 40, 80, and 160 *μ*M) of TBHQ on the viability of rat chondrocytes. (b) Effects of different concentrations (0, 0.625, 1.25, 2.5, 5, 10, and 20 *μ*M) of TBHQ on the viability of rat chondrocytes treated with 20 *μ*M TBHP. Treatment with 2.5 and 5 *μ*M TBHQ had better effects in improving cell viability. ^*∗∗*^*P* < 0.05.

The structural formula of TBHQ is shown in Figure 1(a). In this study, the cytotoxic effects of different concentrations of TBHQ (0, 0.625, 1.25, 2.5, 5, 10, 20, 40, 80, and 160 *μ*M) on chondrocytes were evaluated through the CCK-8 assay. The results showed that TBHQ concentrations equal to or greater than 80 *μ*M were cytotoxic to rat chondrocytes (Figure 2(a)). Moreover, 1.25, 2.5, 5, and 10 *μ*M of TBHQ significantly improved the viability of rat chondrocytes exposed to 20 *μ*M TBHP. Notably, 2.5 and 5 *μ*M of TBHQ had the best effects (Figure 2(b)) and were consequently used in the subsequent in vitro experiments.

### 3.2. TBHQ Prevented Excessive Generation of ROS, Increased the SOD Levels, and Decreased the MDA Levels

The levels of ROS in rat chondrocytes were evaluated through DCFH-DA staining. Flow cytometry analysis demonstrated that treatment with 25 *μ*M of TBHP increased the levels of ROS in chondrocytes, indicating that the oxidative stress model was successfully established. In addition, 2.5 and 5 *μ*M of TBHQ effectively decreased the levels of ROS (Figures 1(b) and 1(c)). The results demonstrated that TBHQ effectively inhibited excessive generation of ROS as a result treatment with THBP. Furthermore, the levels of SOD and MDA were determined to evaluate oxidative damage. The results showed that treatment with THBQ significantly decreased the levels of MDA but increased the levels of SOD in rat chondrocytes exposed to 20 *μ*M TBHP (Figures 1(f) and 1(g)). These suggested that THBQ effectively protected chondrocytes from excessive accumulation of ROS and caused oxidative stress.

### 3.3. TBHQ Prevented Oxidative Stress-Induced Mitochondrial Damage and Cell Apoptosis

**Figure 3:**
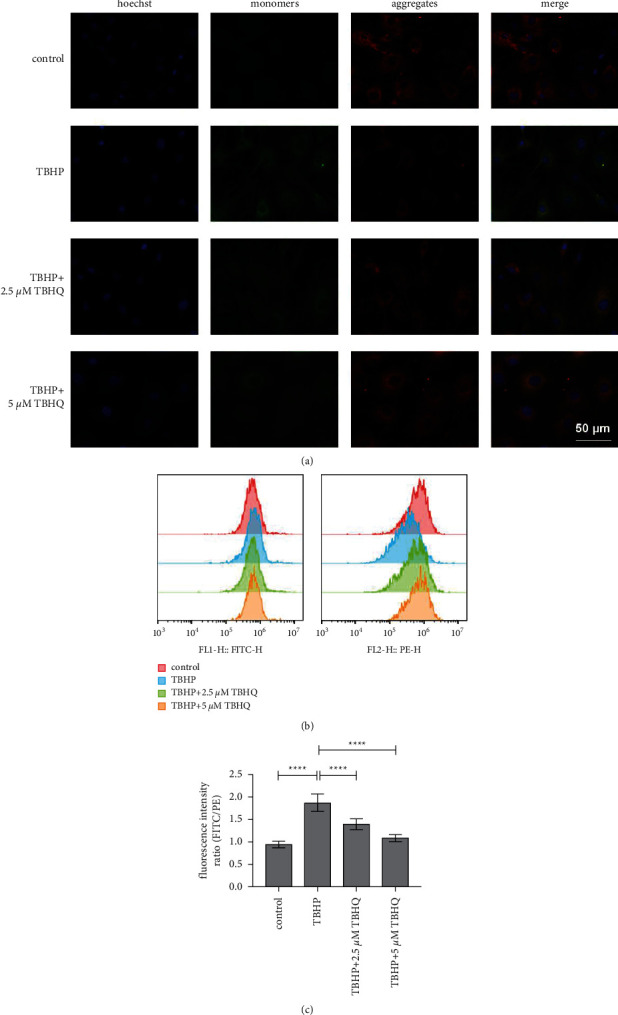
TBHQ prevented oxidative stress-induced mitochondrial damage. (a) Chondrocytes stained with JC-1 and observed under confocal microscopy (magnification: ×200, scalebar: 50 *μ*m). JC-1 aggregates showed red fluorescence, representing normal membrane potential, while JC-1 monomers showed green fluorescence, representing mitochondrial membrane potential depolarization. Blue colour represents the Hoechst-stained cell nuclei. Merge represents a merge of green, red, and blue channels. (b-c) Mitochondrial membrane potential was quantified using flow cytometry. Treatment with 2.5 and 5 *μ*M of TBHQ significantly increased the FITC/PE intensity ratio, compared to treatment with TBHP. This indicated that TBHQ protected rat chondrocytes from mitochondrial damage, induced by oxidative stress. ^*∗∗∗∗*^*P* < 0.01.

The study further used JC-1 staining to evaluate the mitochondrial membrane potential of rat chondrocytes. The monomers (green fluorescence) and aggregates (red fluorescence) were then quantified through flow cytometry. The results showed that the FITC/PE intensity ratio was decreased in two TBHQ treatment groups, compared to that in the TBHP treatment category, indicating that TBHQ protected rat chondrocytes from mitochondrial damage induced by oxidative stress (Figures 3(a) and 3(b)). Furthermore, Annexin V/PI staining was used to determine the apoptosis of rat chondrocytes. The findings showed that treatment with 2.5 and 5 *μ*M TBHQ significantly reduced the number of apoptotic chondrocytes, compared to treatment with TBHP. This suggested that TBHQ protected rat chondrocytes from apoptosis and induced oxidative stress (Figures 1(d) and 1(e)).

### 3.4. TBHQ Ameliorates Oxidative Stress-Induced Matrix Degradation

**Figure 4:**
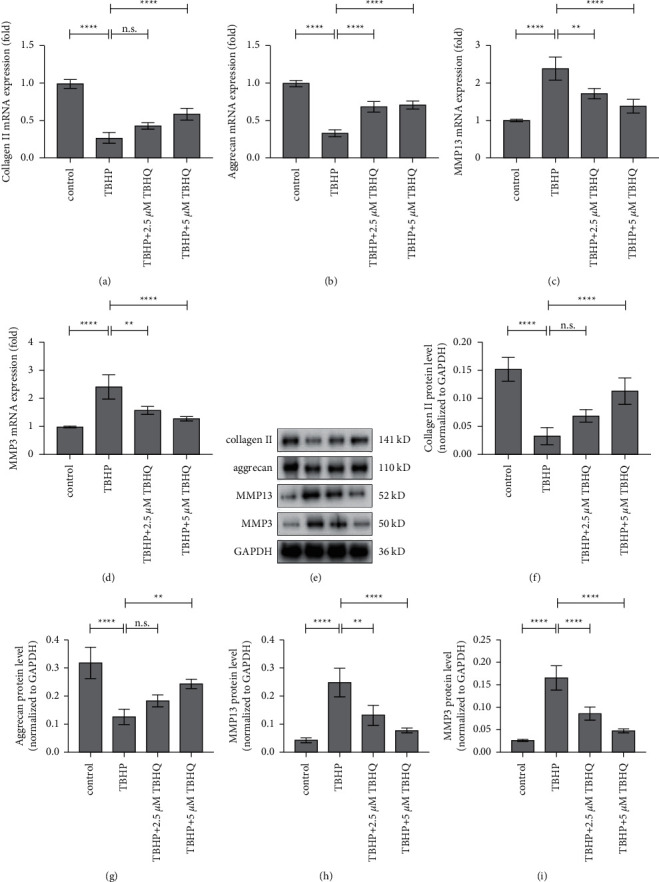
TBHQ prevented oxidative stress-induced matrix degradation. (a–d) The mRNA expression levels of collagen II, aggrecan, MMP3, and MMP13 were evaluated using qPCR. (e) Representative Western blot images of collagen II, aggrecan, MMP3, and MMP13. (f–i) The protein expression levels of collagen II, aggrecan, MMP3, and MMP13 evaluated through Western blotting. ^*∗∗*^*P* < 0.05 and ^*∗∗∗∗*^*P* < 0.01. n.s, not significant.

Western blotting and qPCR were used to evaluate the expression of MMP3, MMP13, aggrecan, and collagen II in rat chondrocytes, at the mRNA and protein levels. The results showed that treatment with TBHQ decreased the mRNA and protein expression levels of MMP3 and MMP13 but enhanced the expression of aggrecan and collagen II in rat chondrocytes treated with 20 *μ*M TBHP (Figure 4). The results therefore demonstrated that treatment with TBHQ ameliorates matrix degradation, induced by oxidative stress, in vitro.

### 3.5. TBHQ Activated the Nrf2 Signalling Pathway

**Figure 5:**
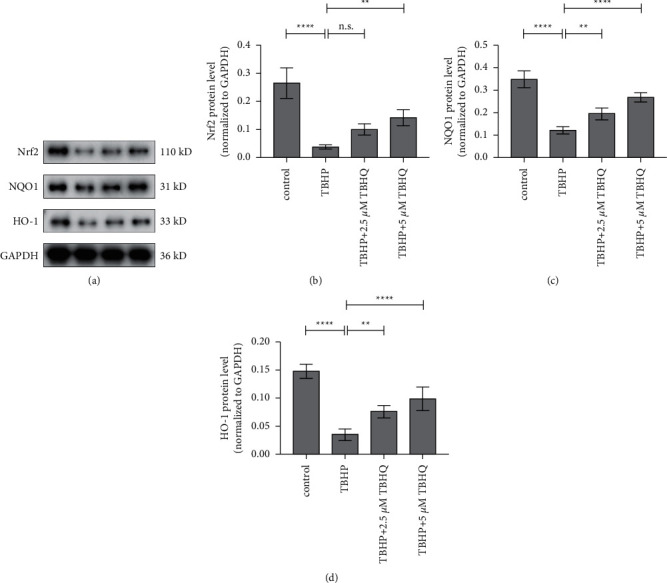
TBHQ activated the Nrf2 pathway in rat chondrocytes. (a) Representative Western blot images of Nrf2, HO-1, and NQO-1. (b) TBHQ significantly increased the protein expression levels of Nrf2 and its downstream molecules, NQO-1 and HO-1. This suggested that TBHQ activated the Nrf2 pathway in rat chondrocytes, in vitro. ^*∗∗*^*P* < 0.05 and ^*∗∗∗∗*^*P* < 0.01. n.s, not significant.

Western blotting was used to measure the expression levels of Nrf2, NQO-1, and HO-1. The results showed that treatment with 2.5 and 5 *μ*M of TBHQ enhanced the expression levels of Nrf2, NQO-1, and HO-1, compared to treatment with TBHP. In addition, the relative expression levels of Nrf2 in the 2.5 *μ*M TBHQ treatment group were higher than those in the TBHP treatment category, although the differences were not statistically significant (Figure 5). The results suggested that treatment with TBHQ activated the Nrf2 pathway and therefore played an antioxidant role in rat chondrocytes.

## 4. Discussion

Osteoarthritis was reported to be strongly associated with increased oxidative stress in chondrocytes. In addition, oxidative damage induces the apoptosis of chondrocytes and degradation of the ECM, which finally leads to degeneration of articular cartilage and contributes to the progression of OA [4, 9]. Moreover, there are currently no reliable drugs to prevent the progression osteoarthritis [2]. Existing research shows that TBHQ is as an effective antioxidant, although little information exists on its benefits in treating osteoarthritis. The present study therefore explored the ability of TBHQ to prevent the apoptosis of chondrocytes and degradation of the ECM, induced by oxidative stress, in vitro.

The findings showed that 2.5 and 5 *μ*M of TBHQ significantly improved the viability of rat chondrocytes treated with 20 *μ*M TBHP. The results from DCFH-DA staining also revealed that 2.5 and 5 *μ*M of TBHQ prevented TBHP-induced excessive production of ROS in chondrocytes. Moreover, treatment with 2.5 and 5 *μ*M of TBHQ reduced the levels of MDA and increased the levels of SOD in rat chondrocytes treated with TBHP. These findings suggested that TBHQ exerted antioxidative effects, in vitro. Notably, decrease in mitochondrial membrane potential is considered a nearly sign of cell apoptosis. Herein, 2.5 and 5 *μ*M of TBHQ prevented the TBHP-induced decrease in mitochondrial membrane potential and dramatically reduced the rate of apoptosis in chondrocytes treated with THBP. Furthermore, PCR and Western blot analyses showed that 2.5 and 5 *μ*M of TBHQ increased the mRNA and protein levels of aggrecan and collagen II but reduced the mRNA and protein levels of MMP3 and MMP9. These findings therefore showed that in addition to preventing cell apoptosis induced by oxidative stress, TBHQ also prevented the degradation of the ECM, caused by oxidative stress. The results from Western blotting also showed that 2.5 and 5 *μ*M of TBHQ enhanced the expression of Nrf2, NQO-1, and HO-1 and decreased the expression levels of Keap1, suggesting that treatment with TBHQ activated the Nrf2 pathway which possibly mediated the beneficial antioxidant effects of TBHQ.

Reactive oxygen species are oxygen-containing free radicals such as *O*_2_^−^, H_2_O_2_, and OH^−^. Additionally, ROS are essential for the maintenance of cellular homeostasis and function [20]. However, excessive accumulation ROS caused by multiple factors may irreversibly damage cells. Increasing evidence also shows that oxidative stress contributes to the pathogenesis of OA. Moreover, dysregulated expression of SOD was observed in the articular cartilages of patients with OA. It was also reported that NO and its derivative could suppress the synthesis of proteoglycan, thus increasing damage to cartilage during the progression of OA [8, 21, 22]. In the present study, the results showed that 2.5 and 5 *μ*M of TBHQ significantly prevented the TBHP-induced apoptosis of rat chondrocytes and ECM degradation in vitro, suggesting that TBHQ can be used as an effective antioxidant to treat OA.

Additionally, Nrf2 is a master regulator of the intracellular antioxidant response. Notably, dissociation of Nrf2 from the Keap-1/Nrf2 complex and the nuclear translocation of Nrf2 are the essential signalling steps for the activation of Nrf2. In addition, the downstream molecules, NQO-1 and HO-1, are often activated to exert antioxidant effects after Nrf2 translocates to the nucleus. The clinical significance of Nrf2 is that it may be used as a pharmacological target, thereby, benefiting patients [11, 12, 23]. Furthermore, chondrocytes are the only resident cell types and the major producers of ROS in articular cartilage. Increased levels of apoptotic chondrocytes were also observed in patients with OA, suggesting that the apoptosis of chondrocytes is crucial in the pathogenesis of OA. Moreover, increase in oxidative stress is positively correlated with collagen degradation, indicating that ROS is an important factor affecting catabolism of the cartilage matrix. Oxidative stress-induced apoptosis of chondrocytes also promotes the expression of matrix-degrading proteases and reduces extracellular ECM synthesis, thus causing ECM degradation. Therefore, preventing the apoptosis of chondrocytes and ECM degradation, induced by oxidative stress, may be a potential therapeutic strategy for OA. In addition, TBHQ is an aromatic organic compound that serves as an Nrf2 activator. It was also reported that TBHQ can protect various cells and organs against oxidative damage by activating Nrf2 signalling. Moreover, the antioxidant properties of TBHQ have been confirmed in patients or animal models of certain diseases [14, 16, 18, 24–28]. The present study demonstrated that TBHQ activated the Nrf2 pathway and upregulated HO-1 and NQO-1 to exert antioxidative effects in rat chondrocytes, consistent with previous reports on other diseases. However, it is not clear whether the Nrf2 pathway is the only signalling pathway through which TBHQ exerts its antioxidant effects. Further research is therefore needed to uncover other possible pathways or validate the present findings.

In summary, the current study showed that TBHQ effectively protected against TBHP-induced oxidative stress, thus inhibiting the apoptosis of rat chondrocytes and degradation of the ECM, by activating the Nrf2 pathway. However, the study only focused on cartilage degeneration. More studies are therefore needed to clarify the effects of TBHQ on synovial inflammation and subchondral bone remodeling. Moreover, further in vivo research is required to establish the effectiveness TBHQ in the treatment of OA. Nonetheless, the study suggests that TBHQ has potential for use in the treatment of OA.

## Figures and Tables

**Table 1:** Primers' sequences used in the real-time PCR (5′-3′).

Name	Forward	Reverse
GAPDH	AAGGTCGGTGTGAACGGATT	TGAGTGGAGTCATACTGGAACAT
MMP 3	GCTCATCCTACCCATTGCAT	GCTTCCCTGTCATCTTCAGC
MMP 13	TGTTTGCAGAGCACTACTTGAA	CAGTCACCTCTAAGCCAAAGAAA
Aggrecan	CTAGCTGCTTAGCAGGGATAACG	GATGACCCGCAGAGTCACAAAG
Collagen II	GGGTCACAGAGGTTACCCAG	ACCAGGGGAACCACTCTCAC

## Data Availability

The data used to support the findings of this study are available from the corresponding author upon request.

## Abbreviations

OA: Osteoarthritis
ROS: Reactive oxygen species
ECM: Extracellular matrix
TBHQ: Tert-butylhydroquinone
Nrf2: Nuclear factor erythroid derived-2-related factor 2
TBHP: Tert-butyl hydroperoxide
MMP: Matrix metalloproteinase
ARE: Antioxidant response element
Keap1: Kelch-like ECH-associated protein 1
NQO-1: NADPH quinone oxidoreductase 1
HO-1: Hemeoxygenase-1
DMEM: Dulbecco's modified Eagle's medium
FBS: Foetal bovine serum
CCK-8: Cell Counting Kit-8
DCFH-DA: 2′,7′-Dichlorodihydrofluorescein diacetate
PI: Propidium iodide
MDA: Malondialdehyde (MDA)
SOD: Superoxide dismutase.
